# Exploring COVID-related relationship extraction: Contrasting data sources and analyzing misinformation

**DOI:** 10.1016/j.heliyon.2024.e26973

**Published:** 2024-02-28

**Authors:** Tanvi Sharma, Amer Farea, Nadeesha Perera, Frank Emmert-Streib

**Affiliations:** Predictive Society and Data Analytics Lab, Faculty of Information Technology and Communication Sciences, Tampere University, Tampere, Finland

**Keywords:** Relation extraction, Deep learning, Natural language processing, Data science, Artificial intelligence, Misinformation, Public health

## Abstract

The COVID-19 pandemic presented an unparalleled challenge to global healthcare systems. A central issue revolves around the urgent need to swiftly amass critical biological and medical knowledge concerning the disease, its treatment, and containment. Remarkably, text data remains an underutilized resource in this context. In this paper, we delve into the extraction of COVID-related relations using transformer-based language models, including Bidirectional Encoder Representations from Transformers (BERT) and DistilBERT. Our analysis scrutinizes the performance of five language models, comparing information from both PubMed and Reddit, and assessing their ability to make novel predictions, including the detection of “misinformation.” Key findings reveal that, despite inherent differences, both PubMed and Reddit data contain remarkably similar information, suggesting that Reddit can serve as a valuable resource for rapidly acquiring information during times of crisis. Furthermore, our results demonstrate that language models can unveil previously unseen entities and relations, a crucial aspect in identifying instances of misinformation.

## 1 Introduction

The COVID-19 pandemic affects billions of people worldwide causing severe damage [1]. Given the novelty of this condition, there are many problems surrounding it, including tracking the spread of COVID-19, estimating the true number of cases, and identifying underlying symptoms [2], [3], [4]. Currently, the most widely recognized symptoms of COVID-19 include fever, fatigue, and shortness of breath [5], [6]. In addition to the previously mentioned symptoms, recent studies have identified other notable manifestations of COVID-19, including gastrointestinal symptoms, such as loss of taste or smell, as well as cognitive impairment and respiratory distress [7], [8], [9], [10]. Furthermore, in clinical and emergency care, uncertainty exists about optimal treatment and medical testing [11].

Given the unprecedented nature of COVID-19, it's unsurprising that no established data repositories were available at the beginning of the pandemic to provide indispensable information for studying COVID-related problems. For this reason, social media platforms have emerged as potential information sources during such emergencies [12], [13], [14], [15]. Consequently, there has been substantial interest in leveraging natural language processing (NLP) methods to extract valuable insights from textual data in this context. This paper aims to contribute to this ongoing effort by delving into the study of relation extraction [16], [17].

Recent progress in deep learning and the introduction of transformer models have changed the landscape of NLP [18]. For example, BERT [19], DistilBERT [20], and BioBERT [21] are all transformer models based on self-supervised learning for building deep neural network models. Such models are trained in two steps. In the first step, unlabeled data are used for the pre-training of the models and then, in the second step, the models are fine-tuned for specific tasks, e.g., for question-answering, relation detection, or classification [22]. In this study, our primary emphasis centers on relation extraction, a process intricately involved in discerning and extracting connections between diverse entities within textual data. An integral initial phase in unraveling these relationships lies in named entity recognition, for which state-of-the-art annotation tools exist.

In general, relation extraction involves analyzing the co-occurrence of entity pairs that represent binary relations [23], [24]. It is worth noting that the terms “relation extraction” and “relation detection” are commonly used interchangeably [25]. However, the task itself can be formulated in various ways. Traditionally, relation detection refers to a binary classification task where the focus is on determining the presence or absence of a relation [26]. In other words, it involves investigating whether a relation exists or not. Nonetheless, this task can be expanded to encompass a multi-class classification approach, which allows for capturing structured relationships and connections between entities within text. This extension permits the specification of relation types beyond mere existence, enabling the categorization of different relation types and providing more detailed information.

In this paper, we explore the relation extraction of COVID-related entities as a multi-class classification task such as disease symptoms (physical and mental symptoms), disorder synonyms, and vaccination types. We examine and contrast the performance of five language models (BERT [19], DistilBERT [20], BioRedditBERT [27], BioBERT [21] and ELECTRA [28]) and identify the data needs for effective learning. What's unique is that we conducted this investigation using two diverse data sources PubMed and Reddit. To make the data suitable for the transformer models, we manually curate large-scale training data. This allows us to contrast the learning behavior of the models in dependence on the data source corresponding to peer-reviewed scientific publications (PubMed) and public discussions of layman (Reddit). Furthermore, we investigate the capability of transformer models to identify novel entities and relations not present in the training data. This is important to make discoveries when the number of synonyms of entities is uncertain for some reason. Lastly, we carry out a systematic analysis to identify misinformation. In the medical field, this is particularly crucial due to the potential impact of false or misleading information on public health and individual decision-making.

The paper's significant contribution is delving into the performance analysis of five pre-trained models sourced from Reddit and PubMed. We specifically focus on the examination of novel predictions, with a crucial emphasis on the detection of “misinformation.” Moreover, we explore the models' prowess in unveiling previously unseen entities, not accounted for in the training data, and assess their effectiveness in identifying instances of misinformation.

This paper is organized as follows. First, we discuss results from related publications and formulate our research questions. Then we introduce all the methods and data we use for our analysis. Thereafter, we discuss our findings and observations for each model. The paper finishes with a discussion and concluding remarks.

## 2 Related work and research questions

In this section, we discuss related work from the literature that is of relevance to our study.

Obtaining data for natural language processing (NLP) experiments related to COVID-19 is challenging due to the novelty of the virus. However, some corpora of scientific publications related to COVID-19 can be used for an NLP analysis, such as those provided by the COVID-19 Open Research Dataset (CORD-19) [29], [30]. Some of these research papers provide automatic labeling for biomedical entity categories, which can be useful for entity recognition and relation extraction tasks [31], [32]. However, currently, for medical texts, there is a severe lack in the availability of labeled datasets preventing the study of supervised learning tasks.

To address this issue, the COVID-19 Annotation and Coding Tool (CACT) has been created to provide a sizable annotated corpus specifically for COVID-19. This tool has been used for NLP to develop models for COVID-19-related tasks, such as symptom identification, disease progression tracking, and treatment recommendation [11].

Regarding the task of relation detection, models like Elmo and BERT [19], [33] demonstrated that the use of an unsupervised pre-training step of language models can significantly improve the performance on various NLP tasks. Typically, the pre-trained is conducted for general domain data, for instance, Wikipedia, while for the fine-tuning of the models domain-specific labeled data are used. Such transfer learning models [34] have become particularly important for scientific NLP where annotated data is limited or expensive to obtain.

For COVID-19-related tasks, domain-specific transformer-based models, such as CovidBERT and CT-BERT [22], [23], trained on a large collection of COVID-19-related literature and social media content, have been introduced. Additionally, to address the lack of high-quality, large-scale labeled scientific data, SCIBERT, a deep learning model based on BERT, was released by [35]. SCIBERT is pre-trained on a multi-domain corpus of scientific publications which makes it domain-independent. As a domain-specific model, BioRedditBERT has been introduced [27]. BioRedditBERT is a BERT model that is pre-trained on large amounts of health-related Reddit posts [27].

For the task of COVID-19-related relation detection, the scientific literature has been utilized in [36] to extract biological mechanisms. Furthermore, in [37], the Transformer-BiLSTM-CRF model has been employed to extract clinical factors and social determinants of health. Additionally, in [38], the RENET2 model was introduced specifically for extracting gene–disease relations from the scientific literature.

While current research has made great progress in applying natural language processing (NLP) approaches to tasks connected to COVID-19, there are still several significant issues and uncertainties that need to be addressed. Our study seeks to address the issues for instance investigating the perceived disparity in text data quality between Reddit and PubMed, particularly concerning relation extraction for COVID-related entities. Within the context of COVID-19, we also aim to assess the predictability of entities previously unseen, using transformer-based models. A crucial aspect that merits further exploration is the effectiveness of these models in identifying false information within COVID-related relationships.

Our research objectives are aligned with the existing gaps in the current literature. Aligned with these objectives, the paper's significant contribution is delving into the performance analysis of five pre-trained models sourced from Reddit and PubMed. It specifically focuses on the examination of novel predictions, with a crucial emphasis on the detection of “misinformation.” Moreover, it explores the models' prowess in unveiling previously unseen entities, not accounted for in the training data, and assesses their effectiveness in identifying instances of misinformation. While transformer-based models have been studied amply for diverse tasks, to our knowledge, we are the first to address these issues systematically.

### 2.1 Research questions

The main research questions addressed in this study can be summarized as follows.

1. Is there a discernible disparity in the quality of text data from PubMed and Reddit concerning the task of relation extraction for COVID-related relations?
2. Can transformer-based models for COVID-related relation extraction predict previously unseen entities?
3. Can transformer-based models for COVID-related relation extraction effectively identify misinformation?

To study the above questions quantitatively, we use 5 transformer-based language models, BERT [19], DistilBERT [20], BioRedditBERT [39], BioBERT [21] and ELECTRA [28], for relation extraction discussed in the following section along with the data used for our analysis.

## 3 Methods

In this section, we discuss the data and models we use for our analysis. We begin with reviewing the data. Then the fine-tuning of the models and their evaluation is discussed. Finally, we describe five language models we use for the COVID-related detection models.

### 3.1 Data

For our analysis, we use the following two sources of data: Reddit and PubMed. In the following, both data sources are discussed in detail.

#### 3.1.1 Reddit data

The dataset from Reddit represents public opinions about COVID-19. We obtained the data by extracting Hot, New, and Top posts from various Reddit pages such as COVID-19, coronavirus UK, coronavirus US, mental health, etc., between January 1, 2020, and April 2021. The entire dataset consists of 25,000 sentences consisting of titles and comments. The total number of words in the dataset is 566,858, with an average word length per sentence of 38 and a total of 2,902,300 characters.

**Figure 1:**
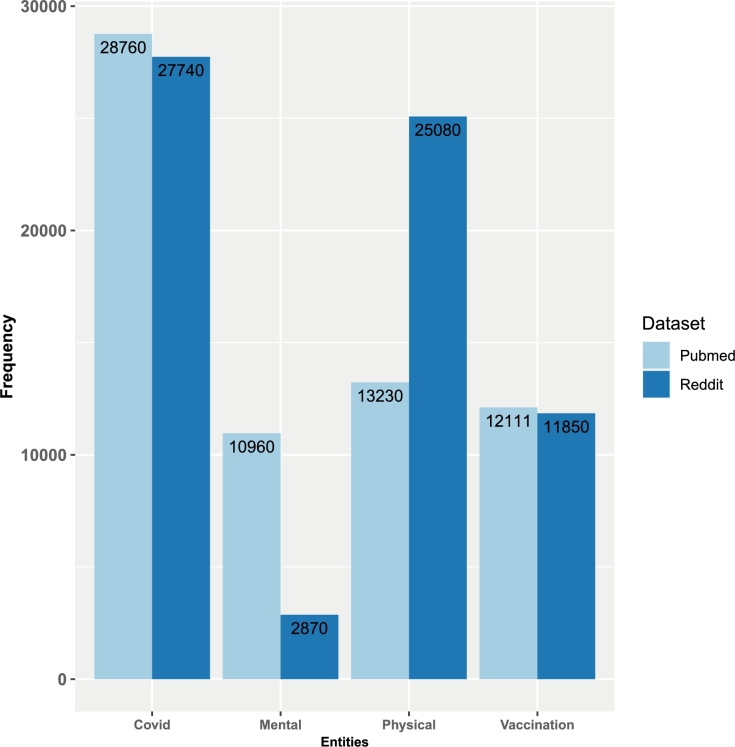
Total number of entity-frequencies of our annotations for COVID synonyms, mental symptoms, physical symptoms, and vaccinations. The color indicates the data source: Reddit (dark blue) and PubMed (light blue).

To make the data usable for our analysis, we performed a manual annotation as shown in Fig. 3 which shows the process of data preparation and annotation of 25,000 sentences for instance Fig. 4 showing how the sentences are annotated. Specifically, we tagged 3 distinct sub-entities/synonyms of COVID (COVID, COVID-19, and coronavirus, refer Table 1). In total, this resulted in 27,740 tagged COVID entities in the Reddit data (see Fig. 1).

**Table 1:** The table presents the sub-entities of ‘COVID’, ‘Physical Symptom’, ‘Mental Symptom’, and ‘Vaccination’ along with their corresponding frequencies in Reddit and PubMed data.

Reddit Data							
COVID		Physical Symptom		Mental Symptom		Vaccination	

COVID	16,060	Fever	50,500	Anxiety	220	Pfizer	5500
Coronavirus	4440	Headache	29,400	Depression	1650	Moderna	3570
COVID 19	8260	Body aches	620	Panic attack	320	Sputnik	250
		Chills	722	Irritation	170	COVAX	170
		Nausea	738	Frustration	270	Astrazeneca	2620
		Dizziness	600	Stress	6100		
		Loss of smell/taste	825	Suicidal thoughts	90		
		Burning sensation	445	OCD	160		
		Vomiting	370				
		Diarrhea	400				
		Shortness of breath	880				
		Sore throat	880				
		Fatigue	2300				
		Cough	4450				
		Flu	2620				
		Sneezing	520				
		Chest heaviness	220				
		Loss of appetite	50				
		Pneumonia	2530				
		Allergy	1180				
		Tiredness	560				
		Lack of sleep	140				

PubMed Data							

COVID		Physical Symptom		Mental Symptom		Vaccination	

COVID	15,560	Fever	4540	Anxiety	1120	Pfizer	7210
Coronavirus	1520	Headache	2530	Depression	420	Moderna	2850
Covid19	10,660	Body aches	380	Panic attack	20	Sputnik	120
		Chills	560	Irritation	50	COVAX	50
		Nausea	500	Frustration	100	Astrazeneca	16,200
		Dizziness	180	Stress	650		
		Loss of smell/taste	1360	Suicidal thoughts	20		
		Burning sensation	250	OCD	250		
		Vomiting	450	Psychiatric diseases	300		
		Diarrhea	200	Psychosis	40		
		Shortness of breath	820	Delirium	100		
		Sore throat	750	Anosmia	50		
		Fatigue	2690	Ageusia	20		
		Cough	5220	Neuromuscular disorder	10		
		Flu	3670	Hypervigilance	20		
		Sneezing	120				
		Chest heaviness	800				
		Loss of appetite	40				
		Pneumonia	1800				
		Allergy	880				
		Respiratory illness	340				
		Lack of sleep	50				
		Cardiopulmonary sequelae	150				
		Arrhythmia	180

Similarly, we tagged 22 unique sub-entities associated with physical symptoms (for instance coughing, pain, shortness of breath, fever, vomiting, diarrhea, chills, headache, sore throat, and more, etc.), totaling 25,080 entities. Additionally, we tagged 8 unique sub-entities linked to mental symptoms (stress, depression, panic attacks, anxiety, irritation, frustration, suicidal thoughts, and obsessive-compulsive disorder (OCD)), with a combined count of 2870 occurrences. Lastly, we labeled 5 unique sub-entities associated with vaccinations (COVAX, Sputnik, Moderna, Pfizer, and AstraZeneca), amounting to a total of 11,850 instances of these entities (see Fig. 1 which shows an overview of the total number of entity frequencies).

#### 3.1.2 PubMed data

The dataset from PubMed represents scientific publications (for instance we searched articles related to COVID-related relation extraction using deep learning) consisting of over 25,000 sentences. The dataset consists of titles and abstracts of articles about COVID and relation extraction. The total number of words used in this dataset is 555,384, with an average sentence length of 45 words and a total of 3,251,377 characters.

From a manual annotation as shown in Fig. 3, we conducted the process of data preparation and annotation of 25,000 sentences. For an example of this annotation process, refer to Fig. 4, which demonstrates how the sentences are annotated.

We tagged 3 unique sub-entities/synonyms of COVID (COVID, COVID-19, and coronavirus) and in total 28,760 tagged COVID entities, 24 unique sub-entities of physical symptoms (headache, fever, body aches, chills nausea, cough and cold, etc.) and in total 13,230 tagged entities, 15 unique sub-entities of mental symptoms (for instance stress, depression, panic attacks, anxiety, irritation, and frustration, etc.) and in total 10,960 tagged entities. Similarly, 5 unique sub-entities of vaccinations (COVAX, Sputnik, Moderna, Pfizer, and Astrazeneca) and in total 12,111 tagged entities; see Fig. 1 for entity frequency and Table 1 for sub-entity frequency.

From Fig. 1, one can see that the Reddit data contain about the same number of COVID entities as the PubMed data and this is also the case for the different types of vaccinations. However, for mental symptoms, there are many more entities in PubMed than in Reddit while for physical symptoms the situation is reversed.

**Figure 2:**
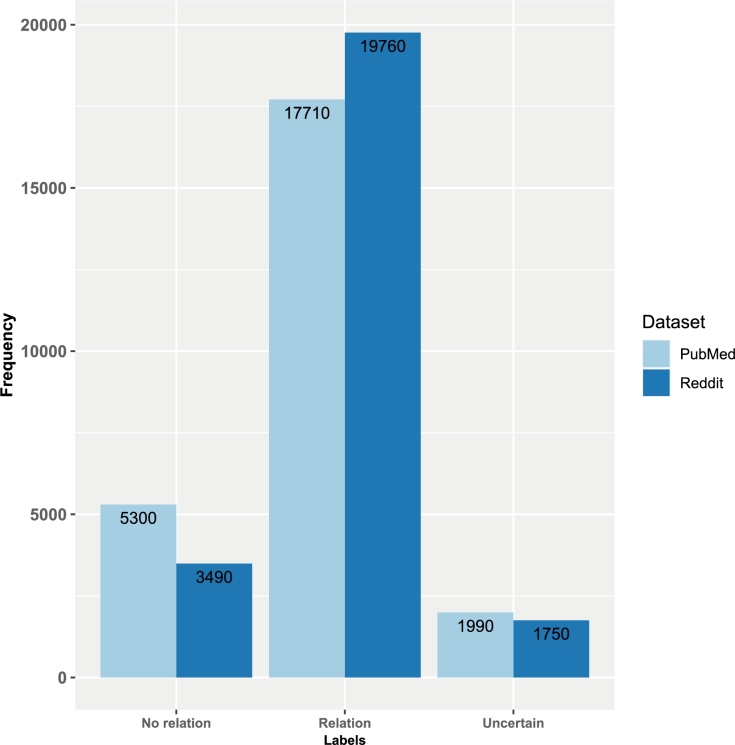
Total number of relation-frequencies of our annotations for relations labeled as “relation”, “no relation”, and “uncertain”. The color indicates the data source: Reddit (dark blue) and PubMed (light blue).

Fig. 2 shows an overview of the number of relations. We annotate also relations between entities. For this, we use the three labels: “relation”, “no relation”, and “uncertain”. In total, we annotate 19,760 relations in Reddit and 17,710 in PubMed, 5,300 “no relations” in PubMed and 3490 in Reddit, and 1990 “uncertain” relations in PubMed and 1750 in Reddit. For our analysis, these labels are used as classes for multi-class classification tasks.

Table 1 showcases the frequency of each sub-entity in both the datasets considered.

### 3.2 Pre-processing of data

**Figure 3:**
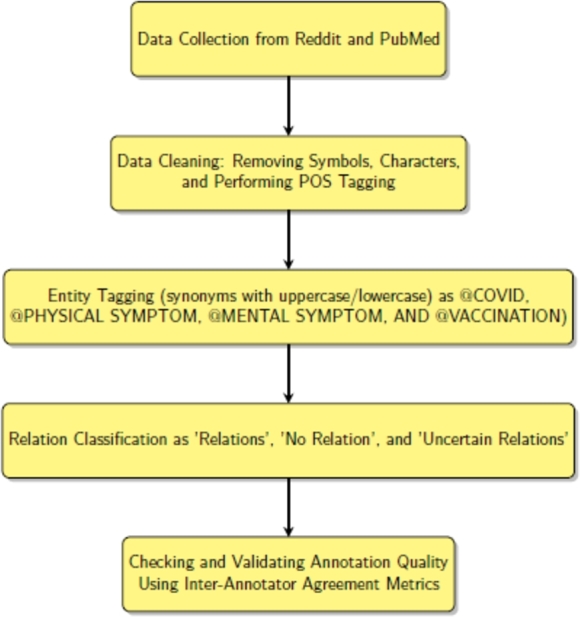
Flowchart showing the process of data preparation and data annotation.

For our analysis, we prepare the data, as shown in Fig. 3. That means, first, we collect data from Reddit and PubMed, manually clean them by removing unwanted symbols, and characters, performing POS tagging, and then tagging them with entities. The text data is then manually annotated with classes such as relation present, no relation, and uncertainty about whether a relation exists. We employ the extracted relations as indicators when deciding whether or not two entities are related in a sentence. Importantly, we consider relation extraction as a multi-class classifying problem because we allow three different labels (“relation”, “no relation” and “uncertain”). The classification problem is solved with one-vs-rest classifiers [24]. Additionally, we use strong measures to guarantee the correctness and uniformity of our annotations, such as evaluating and confirming annotation quality utilizing inter-annotator agreement metrics.

**Figure 4:**
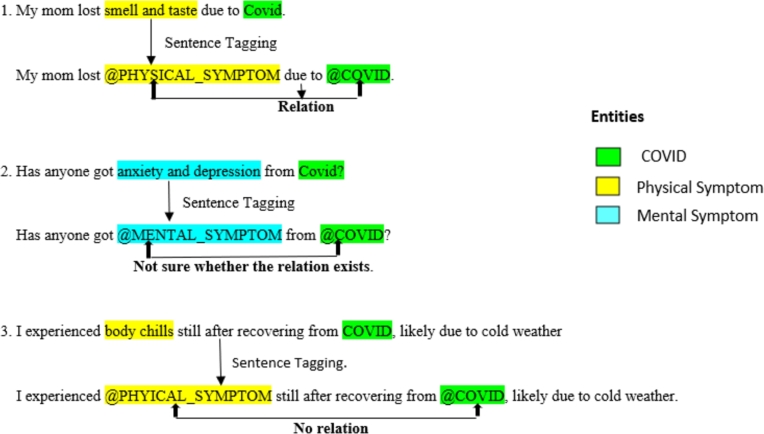
Three example sentences are provided for reference, categorizing whether a relation is present, absent, or uncertain between entities- COVID, symptoms, and vaccinations.

In Fig. 4, example sentences are provided for reference, categorizing whether a relation is present, absent, or uncertain between entities - COVID, symptoms, and vaccinations.

In order to avoid redundancies and typing errors, each of the entities that were selected from the two databases (PubMed and Reddit) was carefully cleaned. Rich text articles are processed, labeled for parts-of-speech (POS) labels, and cleaned semantically [25]. Most of the sentence syntax, including conventional punctuation, is retained after text cleaning since the corpus already has sentence structures that are excellent for parts-of-speech analysis. Only unrecognizable characters and white spaces are eliminated. The two annotated datasets were saved as dot comma-separated values files (CSVs). All the synonyms of COVID with uppercase and lowercase are considered with one tag @COVID. Similarly, physical symptoms, mental symptoms, and vaccination are tagged as @PHYSICAL SYMPTOM, @MENTAL SYMPTOM, and @VACCINATION respectively for instance in Fig. 4.

### 3.3 Description of the models

For our analysis, we use five language models: BERT, BioRedditBERT, ELECTRA, DistilBERT, and BioBERT, refer Table 3. In the following, we describe each of these briefly in terms of architecture, hyper-parameters, speed, and performance.

**BERT:** Bidirectional Encoder Representations from Transformers (BERT) [19] is a recent language representation model that pre-trains a model on a large dataset before fine-tuning the model on another task using a bidirectional transformer network. That means BERT is based on transfer learning. BERT has demonstrated good results on a variety of NLP tasks. However, BERT is costly to compute because of its complicated architecture and size, resulting in slower inferring times. Also, hyper-parameters for BERT include 512 sequence lengths, 12 or 16 attention heads, 768 or 1024 hidden sizes, and 12 or 24 layers.

**DistilBERT:** This model is a light, small, quick, and economical transformer version by distilling BERT [20]. Importantly, DistilBERT maintains nearly 95% of its performance on the GLUE language benchmark while utilizing 40% fewer parameters and operating 60% faster as compared to BERT. The model has a vocabulary size of 30,522 with hyper-parameters - 512 is the maximum number of sequences used, 6 or 12 number of layers, 384 or 512 hidden size, and 6 or 8 attention heads.

**BioRedditBERT:** BioRedditBERT is a BERT model that is initialized with BioBERT and further enhanced through pre-training on health-related Reddit posts [27]. In total, from the beginning of 2015 to the end of 2018 over 800,000 discussions have been collected by crawling through 68 health-themed sub-reddits. To ensure data quality, the dataset underwent cleaning that involved removing deleted posts, comments from bots or editors, and other undesirable content. Finally, the model was trained using a dataset containing approximately 300 million tokens, employing a learning rate of 2e-5 for 100,000 steps. The training was conducted with a batch length a maximum sequence size of 64, and a vocabulary size of approximately 780,000 words. Moreover, given the complexity of the model, BioRedditBERT runs at a speed just like BERT.

**BioBERT:** This is a domain-specific variant of the BERT model called BioBERT (BERT for Biomedical Text Mining) [21]. BioBERT is pre-trained on two large biomedical corpora: PMC full-text articles with 13.5 billion words and PubMed abstracts with 4.5 billion words. Similar in design and hyper-parameters to BERT, the model recognizes biological words, which is difficult for a general language model, because it has prior training in both fundamental and clinical corpus. Similar to BERT in speed, BioBERT has shown results resulting in good performance on several biological NLP tasks in comparison with generalized BERT models.

**ELECTRA:** Efficiently Learning an Encoder that Classifies Token Replacements Accurately (ELECTRA) is a further modification of the BERT model [28]. To increase the efficiency of the pre-training phase, ELECTRA uses an alternate approach for the masked language modeling objective dubbed “replace token detection”. Also, on a variety of NLP tasks, ELECTRA has outperformed BERT while using fewer resources during training. Moreover, in ELECTRA, there are extra parameters associated with the discriminator and generator networks in addition to hyper-parameters which are just like those in BERT.

### 3.4 Fine-tuning of the models

This section shows how the pre-trained models are fine-tuned for instance BERT's fine-tuning is explained below in detail.

• Pre-training: The fine-tuning of pre-trained transformer models is required to optimize a target task. For instance, the transformer models (BERT [19], BioBERT [20], BioRedditBERT [27], DistilBERT [21], and ELECTRA [28]) are pre-trained using a self-supervised learning technique on massive corpora of text data. During this pre-training stage, every word or token in the input sequence can have a contextualized embedding learned by the transformer. Rich representations are generated by the model by capturing the dependencies and relationships between words. Labeled data is utilized to train the pre-trained models on a specific target task which is in our case a multi-class classification task.

• Tokenization and Encoding: Tokenization is the process of dividing the input sequences into words or subwords called smaller tokens with the help of WordPiece tokenization. An exclusive embedding is given to every token after tokenizing the input sequences. By doing this, BERT can process input sequences of varying lengths and comprehend every single word or subword. Tokenization enables the model to capture subtleties in meaning by processing and comprehending the input at every level. Additionally, two special tokens are added at the start and finish of the input sequence which is a separator token that is used to separate the input into multiple segments and a classification token in which the complete input is generated as a fixed-size representation. BERT uses an embedding layer to take the tokenized input and transform it into numerical embeddings. To account for sentence structure, BERT uses positional encoding. Since transformers aren't designed to grasp token order, positional encoding aids BERT in maintaining sequence information.

• BERT's architecture, attention mechanism, and pooled output: Multiple layers of feedforward neural networks and self-attention mechanisms make up BERT. The model performs bidirectional processing on the input sequence, extracting contextual data from the full context for each word. Attention masks are made to distinguish between the legitimate tokens and the padded tokens after trimming the sequences to a predetermined length. As the model processes each token, the attention mechanism allows the model to concentrate more on relevant tokens. It determines the significance of additional tokens towards the representation of the current token by calculating attention ratings. The degree of accuracy of the embeddings is enhanced by attention methods, which assist the model in capturing contextual information and long-range dependencies. Additionally, the last layer's classification token yields a pooled representation. This pooled representation, frequently used in subsequent tasks like classification, serves as a fixed-size embedding for the complete input sequence.

• Task-specific layers and fine-tuning: A task-related layer like the classification layer is put on top of the pre-trained BERT layers by setting the learned weights as the initial values. Then, the labeled data used for the text classification task is used to train the models using gradient descent optimization and backpropagation. Using classified labeled data, the entire model is fine-tuned and while fine-tuning, the pre-trained model layers and the new task-related layers are updated. By adjusting the extracted features of the task, the model is fine-tuned to produce optimal results.

• Model Evaluation: After carefully selecting the hyper-parameters and final fine-tuning, the model's performance is evaluated using error measures (F1 score, accuracy, precision, and recall). Also, we perform a comparative analysis on all models. By estimating learning curves for relation detection, we learn about the predictive performance of the models including associated learning effectiveness regarding the amount of training data [25].

Similarly, the selected pre-trained models undergo similar fine-tuning, each tailored to its unique characteristics. BioBERT, which specializes in biomedical text, undergoes pre-training on a vast biomedical corpus. Employing self-supervised learning tasks, it predicts masked words, enabling BioBERT to grasp contextualized representations specific to biomedical nuances. DistilBERT takes a different route, engaging in a distillation process. Learning from a pre-trained BERT model, DistilBERT aims to transfer knowledge efficiently, resulting in a more compact model that retains substantial representational power. BioredditBERT, on the other hand, is a BERT model that has been pre-trained on large amounts of health-related Reddit posts.

Shifting to ELECTRA, its pre-training strategy diverges from conventional masked language models. Instead of directly predicting masked tokens, ELECTRA introduces an adversarial twist. Some tokens are replaced with incorrect ones, and the model is trained to distinguish between genuine and replaced tokens. This dual-network approach involves a generator and a discriminator, promoting an adversarial training paradigm. By doing so, ELECTRA captures nuanced contextual features and refines its understanding of word relationships. Each model in this suite undergoes a nuanced fine-tuning process, leveraging its distinctive pre-training methodology for enhanced contextual understanding.

### 3.5 Selection of hyper-parameters

The hyper-parameters were meticulously selected and optimized for each model to ensure optimal results. The model's hyper-parameters underwent a systematic selection process involving both manual and automated tuning. Initial hyper-parameter values were set using standard practices. Subsequent experiments, including grid search and random search, assessed performance across various hyper-parameter configurations. The model was trained and evaluated on a validation set for each combination, monitoring metrics like accuracy and F1 score. Experiments adjusted hyper-parameters individually and explored interactions between them. Fine-tuning, guided by initial insights, iteratively refined hyper-parameter choices until a satisfactory combination was identified. For instance, the hyper-parameters were optimized with a learning rate of 2e-5, 3 epochs, and a batch size of 10 instances for the BERT model, refer Table 3. Throughout the training process, the model's performance was assessed for making final predictions. In addition, for fine-tuning the models, we utilized the Hugging Face transformers library, which offers pre-training and fine-tuning capabilities. The fine-tuning procedure involved calculating the loss, accuracy, and F1 score. Tabel 2 shows a summary of all parameters.

**Table 2:** Optimized Hyper-parameters for BERT model.

Parameter	Value
Optimizer	Adam
Batch Size	10
Learning Rate	2e-5
Max Sequence Length	128
Max Epoch	3
Adam Epsilon	1e-8

In addition to it, we chose BERT base as our basis model [22], [40] for further processing as BERT is of two types: BERT base and BERT large. Compared to the BERT basic model, the BERT large requires a lot more memory. The maximal number of iterations for BERT large on a typical GPU with 12 GB RAM is therefore so little that it reduces the accuracy of the model independent of the learning rate [19], [22], [40].

The training was implemented in PyTorch [41] and fine-tuned on a Tesla K80 GPU. Moreover, training on the GPU typically took approximately one to two hours.

### 3.6 Error measures

We compare the models in this study using a variety of error scores [26] for multi-class classification using a 1-vs rest approach [24], [42]. Specifically, we are using: accuracy, precision, recall, F-score, and AUC-ROC, refer Table 3. The error scores for multi-class classification problems are computed separately for each class, namely “relations present,” “no relations,” and “uncertain relations.” These individual class scores are then averaged using the macro-averaging approach to obtain an overall score [42]. For instance, first, the accuracy of classes - ‘Relations’, ‘No relations’, and ‘Uncertainty’ is calculated and then the average accuracy of all the classes is calculated. Similarly, the average for precision, recall, and F score is calculated. The following are the standard error scores for a binary-class classification upon which the averages are based:

(1) $$\text{Accuracy}=\frac{\text{TP + TN}}{\text{TP + TN + FP + FN}}$$

(2) $$\text{Precision}=\frac{\text{TP}}{\text{TP + FP}}=\frac{\text{Relevant Relations Recognized}}{\text{Total Relations Recognized}}$$

(3) $$\text{Recall/ Sensitivity/ TPR}=\frac{\text{TP}}{\text{TP + FN}}=\frac{\text{Relevant Relations Recognized}}{\text{Relevant Relations in Data}}$$

(4) $$\text{False Positive Rate (FPR)}=\frac{\text{FP}}{\text{TN + FP}}$$

(5) Specificity=1 - FPR

(6) $$\text{F-score}=\text{2}\cdot \frac{\text{Precision ⋅ Recall}}{\text{Precision + Recall}}$$

where accuracy (see Eqn. (1)) is the ratio of cases—true positives and true negatives, that were accurately predicted relative to all instances. Precision (see Eqn. (2)) is detecting pertinent relations indicated by the ratio of true positives to the sum of true positives and false positives. In addition, Recall/Sensitivity/TPR (see Eqn. (3)) is the model's capacity to identify important relations among all the relevant relations in the data is indicated by the ratio of true positives to the sum of true positives and false negatives. The false positive rate (FPR) (see Eqn. (4)) is the rate of inaccurate positive predictions expressed as the ratio of false positives to the total of true negatives and false positives. Also, specificity (see Eqn. (5)) measures how well the model avoids false positives and is computed as 1 minus the False Positive Rate. F-score (see Eqn. (6)) is a balance between recall and precision is achieved by combining the two metrics. Additionally, TP stands for True Positives, TN for True Negatives, FP for False Positives, and FN for False Negatives. Here, recall is the sensitivity or the true positive rate (TPR) of the model. Whereas the specificity is (1 - FPR).

**Table 3:** Experimental setup for the multi-class classification task.

Experimental environment	Description
Task	Relation extraction (Multi-class classification)
Models	BERT, BioBERT, BioRedditBERT, DistilBERT, ELECTRA
Data sources	Reddit and PubMed
Pre-training	Already pre-trained models on diverse sources
Fine-tuning	Manually annotated data from Reddit and PubMed
Evaluation metrics	Accuracy, F1-score, Precision, Recall, AUC
Hardware	Tesla k80 GPU and 12 GB RAM
Software	PyTorch
Model hyperparameters	Optimizer, Batch size, Learning rate, Epoch, Max sequence length and Adam epsilon
Evaluation criteria	10-fold cross-validation

### 3.7 Model evaluation and assessment

For our analysis, we use a 10-fold cross-validation (CV) [22]. Cross-validation is a re-sampling method [43], [44], [45] in which the data for parameter estimation and model evaluation are repeatedly divided into training and validation set. Importantly, test data re-acquired during this process is used to evaluate the model [46]. A model evaluation is a method of assessing or evaluating the performance of the model. For the model assessment, we sub-sample and re-sample the data. In sub-sampling, the data are divided into 10 random sub-samples of the data set, and each sub-sample is re-sampled to evaluate the performance of the model. For model selection, a cross-validation strategy (CV) is used [43], [47], [48], [49].

In addition, we assess the models' performance through error scores. However, relying solely on error scores does not conclusively determine which model performed well based on their classification errors. For a robust evaluation, it is crucial to statistically examine whether observed performance differences hold significance. To address this, we employed a non-parametric statistical test [50], the Mann-Whitney U test. This test is apt for situations where parametric test assumptions, such as those of the t-test, are not met, such as in cases of limited sample sizes, non-normally distributed data, or unequal variances. The Mann-Whitney U test assesses whether distributions of two independent samples significantly differ. Hence, rejection of the Null hypothesis indicates a significant difference in the distributions.

Table 3 provides a comprehensive overview of our experimental environment along with their respective descriptions. This table serves as a summary encapsulating the key elements of our experimental setup.

## 4 Results

In this section, we present numerical results for five language models, BERT, BioRedditBERT, ELECTRA, DistilBERT, and BioBERT, for the analysis of text data from Reddit and PubMed. First, we analyze the data and assess the quality of the models by evaluating their learning curves. Then we assess the runtime of the models, evaluate their overall performance, and compare their results. Finally, we study the prediction of new entities and present results for the quantification of misinformation.

### 4.1 Quality of models and data

A learning curve represents the performance of a model for different sample sizes of the training data [51], [52]. Learning curves enable model diagnostics by providing an answer to the question of how much data is required to achieve a given performance. Furthermore, by comparing multiple learning curves from different models, one can assess differences in models regarding their learning behavior.

In general, the size of the training data and its quality have a direct impact on the performance of the models. In this analysis, we study the effects of the data quality and quantity of the five models on error scores assessed with data from Reddit and PubMed. Specifically, we use the five models BERT, BioRedditBERT, ELECTRA, DistilBERT, and BioBERT, and train each model separately with data from Reddit and PubMed. For each run, we use a certain amount of training samples, e.g., 2%, 4%, 7%, 10% to 100% of training data where 100% training data correspond to 22500 samples with a test data size of 2500. To obtain reliable estimates, we averaged over ten independent runs and performed 10-fold cross-validation for each dataset. The samples of the training data were chosen through a systematic sampling approach. Specifically, we employed a random sampling technique to ensure a representative selection from the entire dataset. Random sampling helps mitigate biases and ensures that each data point has an equal chance of being included in the subset. This approach allows us to draw meaningful conclusions about the model's performance across different proportions of the training data, providing insights into its scalability and generalization capabilities.

**Figure 5:**
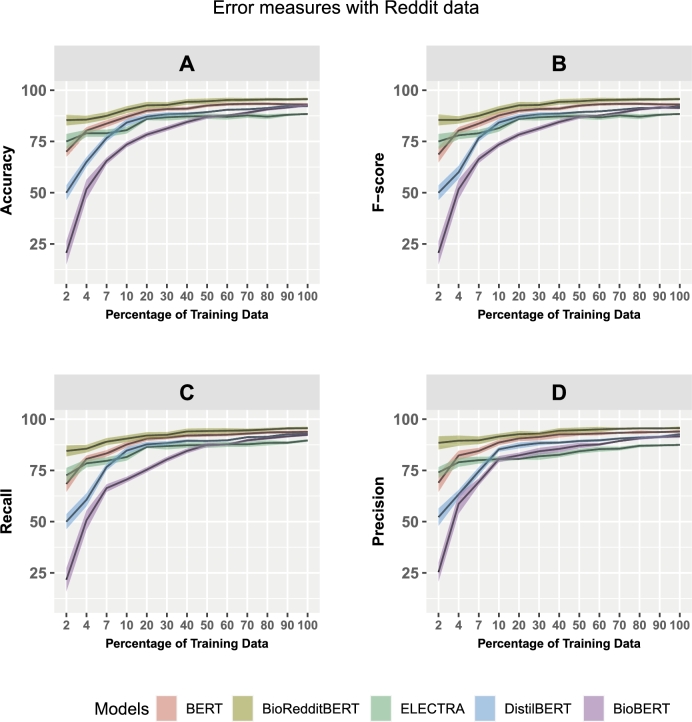
Learning curves show the model performance in dependence on the size of the training data. Error measures: accuracy, F score, recall, and precision. The panel refers to the models: BERT, BioRedditBERT, ELECTRA, DistilBERT, and BioBERT. The data are from Reddit.

**Figure 6:**
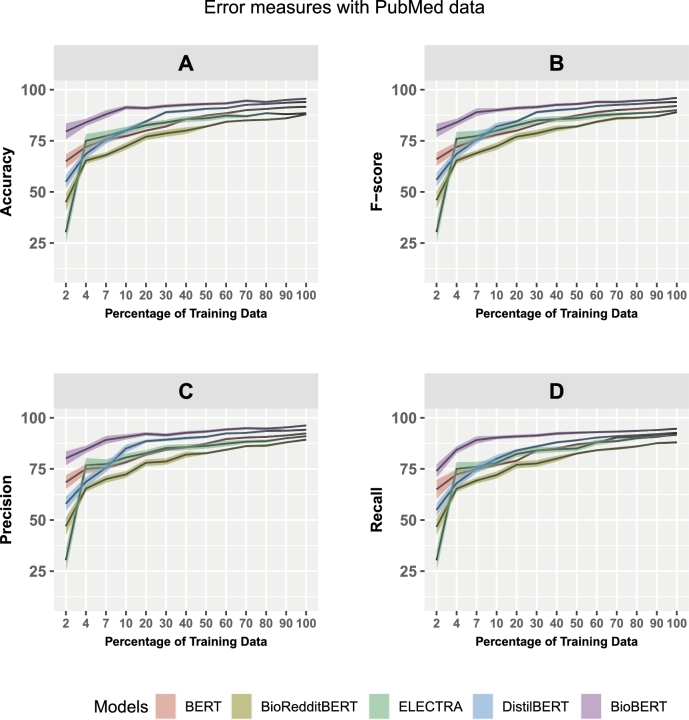
Learning curves show the model performance in dependence on the size of the training data. Error measures: accuracy, F score, recall, and precision. The panel refers to the models: BERT, BioRedditBERT, ELECTRA, DistilBERT, and BioBERT. The data are from PubMed.

In Figure 5, Figure 6, we show the learning curves of the five studied models. These figures show the accuracy (A), F-score (B), precision (C), and recall (D) for Reddit (Fig. 5) and PubMed (Fig. 6). The shaded areas in the learning curves represent the standard errors, and the black lines show the mean values of the corresponding scores.

In Fig. 5 and Table 4 we show the performance of the models for 90% of the training data. From these results one can see that BioRedditBERT is the best model for all four error measures and all sizes of the training data. This determination, based on error measures, is further supported by a rigorous statistical analysis using the Mann-Whitney U test, establishing BioRedditBERT's statistical significance compared to the other models. Also, its standard error is lower than for the other models (Table 4). On the other hand, BioBERT is the worst model for training data below 50%, yet displays improvement with larger datasets. Overall, all five models converge with an increasing size of the training data indicating that a sufficient amount of data is available. Furthermore, all five models achieve a reasonable performance indicating that the quality of the data is sufficient to fit the models. In summary, our comprehensive evaluation, combining error scores and statistical significance, shows BioRedditBERT as the superior model for the Reddit data.

**Table 4:** Analysis results of the five models for Reddit data evaluated using 10-fold CV. The size of the training data was 90%. Here S.E. corresponds to the standard error. A: Results for the error measures. B: Results for a two-sample Mann-Whitney U test for a pairwise comparison of models. P-values less than 10^−4^ are indicated by a ‘*’.

A: Model	Accuracy		F Score		Recall		Precision	
	Mean	S.E.	Mean	S.E.	Mean	S.E.	Mean	S.E.
BERT	0.885	0.108	0.885	0.122	0.886	0.118	0.891	0.138
BioRedditBERT	0.923	0.136	0.923	0.135	0.920	0.136	0.931	0.134
ELECTRA	0.845	0.141	0.845	0.142	0.847	0.146	0.827	0.123
DistilBERT	0.835	0.113	0.829	0.116	0.834	0.117	0.833	0.126
BioBERT	0.764	0.160	0.764	0.161	0.760	0.162	0.787	0.165

B: Model	Accuracy		F Score		Recall		Precision	
	*p* value	*p* < 0.05	*p* value	*p* < 0.05	*p* value	*p* < 0.05	*p* value	*p* < 0.05

BioRedditBERT vs BERT	0.045	Yes	0.045	Yes	0.049	Yes	0.044	Yes
BioRedditBERT vs ELECTRA	0.001	Yes	0.001	Yes	0.003	Yes	0.0014^⁎^	Yes
BioRedditBERT vs DistilBERT	0.005	Yes	0.004	Yes	0.008	Yes	0.007	Yes
BioRedditBERT vs BioBERT	0.001	Yes	0.001	Yes	0.001	Yes	0.001^⁎^	Yes

Fig. 6 and Table 5 show the learning curves and analysis results for PubMed data. For this BioBert is the best-performing model for all error measures and all sizes of training data. This determination, based on error measures, is further supported by a rigorous statistical analysis using the Mann-Whitney U test, establishing BioBERT's statistical significance compared to the other models. Interestingly, now BioRedditBERT is the worst model. Specifically, BioBERT achieves the highest accuracy of 91.07 with a lower standard error (0.89) and BioRedditBERT achieves the lowest accuracy of 76.68 with a standard error (of 1.26) compared to the other models, i.e., BERT (83.10), ELECTRA (80.01), and DistilBERT (84.39).

**Table 5:** The results of the five models for PubMed data evaluated using 10-fold CV. The size of the training data was 90%. Here S.E. corresponds to the standard error. A: Results for the error measures. B: Results for a two-sample Mann-Whitney U test for a pairwise comparison of models. P-values less than 10^−4^ are indicated by a ‘*’.

A: Model	Accuracy		F Score		Recall		Precision	
	Mean	S.E.	Mean	S.E.	Mean	S.E.	Mean	S.E.
BERT	0.828	0.109	0.831	0.103	0.827	0.103	0.839	0.103
BioRedditBERT	0.766	0.126	0.772	0.129	0.770	0.124	0.777	0.128
ELECTRA	0.800	0.163	0.804	0.171	0.803	0.166	0.807	0.175
DistilBERT	0.843	0.099	0.847	0.003	0.832	0.102	0.855	0.110
BioBERT	0.910	0.089	0.911	0.089	0.900	0.097	0.915	0.091

B: Model	Accuracy		F Score		Recall		Precision	
	*p* value	p < 0.05	*p* value	*p* < 0.05	*p* value	*p* < 0.05	*p* value	*p* < 0.05

BioBERT vs BERT	0.002	Yes	0.003	Yes	0.004	Yes	0.001	Yes
BioBERT vs BioRedditBERT	0.001^⁎^	Yes	0.001^⁎^	Yes	0.001^⁎^	Yes	0.001^⁎^	Yes
BioBERT vs ELECTRA	0.001	Yes	0.001^⁎^	Yes	0.001	Yes	0.001^⁎^	Yes
BioBERT vs DistilBERT	0.048	Yes	0.048	Yes	0.014	Yes	0.044	Yes

Table 5 shows the results for 90% of the training data of the error measures: Accuracy, F-Score, Recall, and Precision assessed with PubMed data. BioBERT achieves the highest accuracy, F-score, recall, and precision, whereas BioRedditBERT is the worst-performing model.

Interestingly, we can see that there is a large difference in the performance of the five models between the two data sets. For Reddit data, Fig. 5 A-D, BioRedditBERT and BERT perform well with F1 scores of 92.34 and 88.50, respectively (Table 4), compared with PubMed data, Fig. 6 A-D: BioBERT (F score 91.13) and DistilBERT (F score 84.73) perform well (Table 5). In addition, ELECTRA and DistilBERT achieve an average accuracy of 84.51 and 82.99, respectively, on Reddit data (Table 4), whereas ELECTRA (F-score 80.43) and BERT (F-score 83.10) achieve average results on PubMed data (Table 5). On the other hand, BioBERT with an F-score of 76.43 on Reddit data (Table 4) and BioRedditBERT with an F-score of 77.22 on PubMed data (Table 5) do not perform as well compared with the other models.

### 4.2 Runtime of models

For assessing models, not only their performance is important but also their runtime. For this reason, we study the runtime for the five models. The results of this analysis are shown in Table 6.

**Table 6:** Run times of BERT, BioRedditBERT, ELECTRA, DistilBERT, and BioBERT for Reddit and PubMed data. The size of the training data is 100%.

	Total training time	
Model	Reddit	PubMed
BERT	52 m 24 s	49 m 00 s
BioRedditBERT	50 m 43 s	41 m 55 s
ELECTRA	29 m 09 s	29 m 33 s
DistilBERT	24 m 15 s	35 m 11 s
BioBERT	44 m 41 s	49 m 31 s

From Table 6 one can see that DistilBERT and ELECTRA are overall fastest compared to the other models. This is plausible because DistilBERT has been introduced as a lightweight version of BERT. This is clearly reflected in the runtimes because, for Reddit data, DistilBERT is more than twice as fast as BERT. Interestingly, the underlying data have an impact on the runtime too, as can be seen from comparing the results for the PubMed data. For these data, ELECTRA is even faster than DistilBERT.

### 4.3 Overall evaluation of the models

Next, we study the evaluation of the models using all training data.

**Figure 7:**
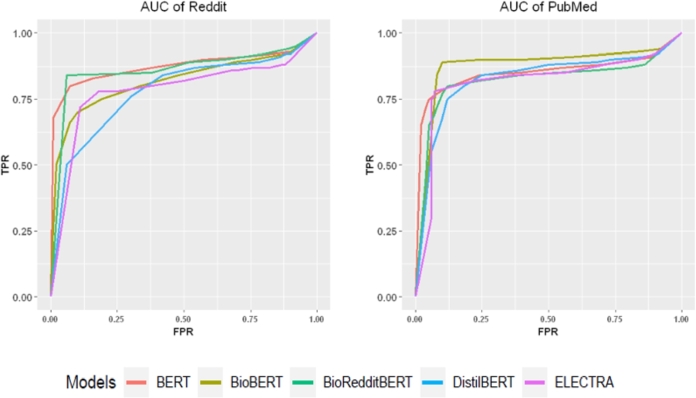
ROC curves of five models. The panel refers to the models: BERT, BioRedditBERT, ELECTRA, DistilBERT, and BioBERT. Data are from Reddit (left) and PubMed (right).

In Fig. 7, we show the ROC (receiver operating characteristic curve) for the overall performance of the five language models. The AUC value (area under the curve) of the ROC curve shows how the five models perform. From Fig. 7 one can see that all models perform reasonably well and from Table 7, we see that BioRedditBERT is best for the Reddit data and BioBERT for the Pubmed data.

**Table 7:** Average AUC values of the five language models for Reddit and PubMed data for all three classes i.e. ‘relation’, ‘no relation’, and ‘uncertainty’.

Model	AUC (Reddit)	AUC (PubMed)
BERT	0.873	0.854
BioRedditBERT	0.862	0.822
ELECTRA	0.783	0.817
DistilBERT	0.781	0.827
BioBERT	0.822	0.884

Table 8 shows the FPR (False Positive Rate) which is - specificity and TPR (True Positive Rate) values of the models for Reddit (first column) and PubMed (second column). One can see that BioBERT and BioRedditBERT achieve lower FPR and TPR for Reddit and PubMed data, respectively. Similarly, BioRedditBERT and BioBERT achieve higher FPR and TPR with Reddit and PubMed data, respectively.

**Table 8:** Mean FPR (false positive rate) and TPR (true positive rate) of the models for the Reddit and PubMed data.

	Reddit		PubMed	
Model	Mean FPR	Mean TPR	Mean FPR	Mean TPR
BERT	0.602	0.782	0.440	0.831
BioRedditBERT	0.702	0.862	0.336	0.732
ELECTRA	0.612	0.796	0.456	0.761
DistilBERT	0.598	0.785	0.502	0.787
BioBERT	0.436	0.721	0.554	0.846

In summary, the results for the AUC demonstrate that PubMed data, i.e., scientific publications, give very similar results compared to Reddit data, i.e., public opinions, for predicting COVID-related relationships.

### 4.4 Comparison of results from Reddit and PubMed

So far, we analyzed the models for the Reddit and PubMed data separately. Now, we conduct a close comparison of the entities and relations obtained in Reddit and PubMed. This will allow us to gain further insights into the differences between a “public discussion” (Reddit) and a “scientific discussion” (Pubmed).

#### 4.4.1 Similarities between Reddit and PubMed

We start by comparing the frequency of entities found in PubMed and Reddit. For this, we are comparing the correlation of vectors using Spearman's rank correlation coefficient [53]. We are using Spearman's rank correlation and not Pearson correlation because we are interested in the ordering of the entities and not their absolute frequencies. Due to the fact that for COVID and vaccination entities, we have only 3 respectively 5 different entities (see Section 3.1), we limit the following analysis to entities for physical symptoms (22 unique entities) and mental symptoms (8 unique entities).

Spearman's rank correlation coefficient, *ρ*, see Eqn. (7), is estimated as follows:

(7) $$\rho =1-\frac{\sum_{i}d_{i}^{2}}{n(n^{2}-1)}$$

where $d_{i}$ is the rank difference, and *n* is the number of different entities. Spearman's rank correlation is separately estimated for the categories (A) physical symptoms and (B) mental symptoms, each giving a correlation value for comparing the ordering of frequencies from PubMed and Reddit. In order to assess the statistical significance, we use a non-parametric Spearman's rank correlation test [50], [53].

Table 9 shows Spearman's rank correlation coefficients (*ρ*) and p-values obtained for the entities: physical symptoms and mental symptoms. The numbers in brackets give the number of unique entities. The correlation and p-value for the entity “physical symptoms” are 0.94 and 6.7e-11 respectively, showing that there is a very strong positive association between Reddit and PubMed. For a significance level of α=0.05, we reject the null hypothesis of no correlation and find that this correlation is statistically significant. For “mental symptoms”, we find a correlation and p-value of 0.71 and 0.04, respectively. This means that also this correlation is statistically significant, but much weaker than for “physical symptoms”.

**Table 9:** The Spearman's rank correlation coefficients and p-values of the entities: Physical symptoms and mental symptoms.

Entity	Spearman Correlation	P-value
Physical Symptoms (22)	0.948	6.7 × 10^−11^
Mental Symptoms (8)	0.718	0.044

We want to remark that also for the entities from the categories “COVID” and “vaccination” between Reddit and PubMed, we obtain statistically significant results, even with a perfect correlation. However, this is less informative than for the above entities due to the very small number of unique entities in these categories.

#### 4.4.2 Prediction of new entities and relations

Next, we take a closer look into so-called “newly predicted entities”. To do this, we check all predicted relations indicated as false positives. In case these relations contain entities that make sense for a given entity category, we call these “newly predicted entities”.

In the following, we list all newly predicted entities. The numbers in brackets give the observed frequencies.

• **COVID:**
  Pubmed: SARS-COV-19 (80)
  Reddit: Covidday (1), Covidgrief (1), Covidlonghualers (30), Covidsmell (1), Covids (1)
• **Physical Symptoms:**
  Pubmed: Palpitations (1), joint pain (3), chest pain (4), tightness (1), heart failure (1), myocarditis (1), Venous thromboembolic disease (1),
  Reddit: Palpitations (6), joint pain (7), chest pain (41), tightness (14), heart failure (1), myocarditis (3)
• **Mental Symptoms:**
  Pubmed: Loss of memory (1), hypotension (1), brain fog (5)
  Reddit: Brain fog (150)
• **Vaccination:**
  Pubmed: None
  Reddit: Sinovac (4), Johnson and Johnson (33)

It is interesting to see that the above predictions are all correct, except for the entities (Covidday, Covidgrief, Covidlonghualers, Covidsmell, Covids) in the Reddit data under the category ‘COVID’. These particular entities do not hold significant meaning and are not commonly used when referring to COVID. It seems these terms reflect colloquial expressions used in casual settings.

More importantly, the remaining predictions mentioned above are indeed accurate and meaningful when it comes to physical symptoms, mental symptoms, and vaccinations. The new entities associated with physical symptoms and mental symptoms from both Reddit and PubMed are relevant and can be considered as actual physical and mental symptoms of COVID. Similarly, for the entity ‘vaccination’, on Reddit we find two new entities, namely “Sinovac” and “Johnson and Johnson,” which are recognized COVID vaccines. Hence, all of these predictions are indeed newly predicted entities.

For our next analysis, we investigate in what relations these novel entities appear. Specifically, there are three possibilities that can occur for a relation: (1) both entities are new, (2) only the first entity is new, and (3) only the second entity is new. The number in the bracket shows the number of relations we find for the corresponding cases.

1. New entity - New entity: PubMed (5), Reddit (None)
2. New entity - Known entity: PubMed (19), Reddit (24)
3. Known entity - New entity: PubMed (25), Reddit (32)

From this, we can conclude that there is also a larger number of predicted relations that are indeed correct. Some examples of correct relations are:

1. The patients reported low exposition to SARS-COV-19, but one case was confirmed with palpitations.
2. Infection of humans with SARS-COV-19 virus causes a disease with symptoms ranging from asymptomatic to severe pneumonia.
3. Pooled prevalence data showed the 10 most prevalent reported COVID-19 symptoms were fatigue, shortness of breath, joint pain, altered smell, altered taste and diarrhoea.

### 4.5 Quantification of misinformation

The last study we conduct augments our analysis by introducing a new category for a relationship. So far we used three categories: “relation”, “no relation”, and “uncertain”. Now, we add one new category called “misinformation” making it a four-class classification task.

We introduce the new label, “misinformation” to identify sentences that appear to have a relation but actually convey false information. We study this only for Reddit because PubMed is a standardized scientific repository containing exclusively peer-reviewed information. For this reason, one would not expect misinformation to be present in published articles. In contrast, Reddit is a social media platform open to essentially anyone. For this reason, the reliability of the available information is uncertain, and there is even a possibility of false information being present. For example, “COVID-19 causes severe rashes and skin discoloration all over the body”, would fall under this category, as it is factually incorrect, given current knowledge.

In order to investigate the presence of misinformation in predicted relations, we create new training data that include also sentences with false relations. Specifically, we gathered 1500 in additional sentences from Reddit that have relations categorized as “misinformation”. That means, we collected sentences that appear to indicate a relationship but actually convey false and misleading information. We combined these data with our previous data from Reddit and re-trained the language models.

In the following, we give a few examples showing misinformation extracted from Reddit as used in our training data:

1. Excessive hair loss is one of the primary signs of COVID-19.
2. Hey guys, just wanted to share that a new symptom of COVID was discovered - purple spots on the skin, so be careful and try if you are experiencing this symptom.
3. COVID-19 causes widespread skin discolouration.

Based on our prior analysis, which indicated that ELECTRA and DistilBERT offer a commendable balance between speed and performance, we focus exclusively on these models for the subsequent analysis. The results for these models are shown in Table 10, Table 11. The AUC values indicate that both models exhibit comparable performance in learning the 4-class classification problem as they do for the 3-class classification problem (see Table 7). Furthermore, Table 11 shows that the individual F-scores for the 4 classes are balanced indicating that each class is learnable to a high extent. Specifically, the F-score for “misinformation” using the ELECTRA model is 82.1% and 84.2% for DistilBERT indicating that the two models are capable of categorizing occurrences of misinformation effectively. Finally, the macro-averaged F-scores for ELECTRA and DistilBERT show an overall good performance demonstrating that the detection of misinformation can be considered as a classification problem which means it is learnable.

**Table 10:** The AUC, FPR, and TPR values for ELECTRA and DistilBERT for the 4 class classification problem containing the class ‘misinformation’. Data are from Reddit.

Model	AUC	False Positive Rate (FPR)	True Positive Rate (TPR)
ELECTRA	0.796	0.536	0.816
DistilBERT	0.808	0.640	0.832

**Table 11:** Individual F-scores and macro-averaged F-scores for the two models ELECTRA and DistilBERT trained on Reddit data including misinformation. The number of predictions for each class is given in brackets, indicating the count for a specific relation. Data are from Reddit.

Model	F-score				Macro F-score
	Relation	No Relation	Uncertainty	Misinformation	
ELECTRA	0.880 (1733)	0.783 (272)	0.804 (140)	0.821 (128)	0.822
DistilBERT	0.900 (1772)	0.800 (292)	0.821 (143)	0.842 (132)	0.841

Finally, we investigate the newly identified entities found in relations corresponding to misinformation; similar to our analysis conducted above (see Sec. 4.4.2). Again, there are three possibilities that can occur in such a relationship. The following shows the observed possibilities with the number of their occurrences in brackets.

1. New entity - New entity: Reddit (None)
2. New entity - Known entity: Reddit (2)
3. Known entity - New entity: Reddit (12)

In the following, we give three examples showing newly identified misinformation.

1. In December, my wife had excessive bleeding during her pregnancy and she was diagnosed with COVID.
2. My mother experienced rashes on her legs when she suffered from COVID.
3. According to what my uncle told me, COVID always results in chest pain irrespective of your age.

The first two examples clearly have no symptoms of COVID-19 while the third one is a generic symptom also not known as a typical symptom for COVID-19 [54], [55], [56], [57].

## 5 Discussion

Conducting studies on COVID-related issues present significant challenges due to the novelty of the disease and the limited availability of well-established benchmark datasets specifically designed to address particular research questions. This becomes especially challenging when employing supervised learning methods for natural language processing (NLP) tasks. Therefore, in this study, we manually annotated a large-scale dataset (see section 3.1) allowing us to delve into transformer models and explore specific questions related to relation detection and extraction.

For our analysis, we selected 5 well-established transformer-based models - BERT [19], DistilBERT [20], BioRedditBERT [39], BioBERT [21] and ELECTRA [28] - as language models for the detection of relationships between COVID-related entities because such deep learning models have been shown to perform superior compared to traditional NLP approaches [58]. Furthermore, our selection of the five models was purposeful, considering their pre-training on both biomedical corpora and Reddit content. This intentional choice is integral to our research, as it harmonizes seamlessly with the dual nature of our data, encompassing both biomedical text and Reddit content. On a technical note, we want to remark that the studied relation extraction task is realized as a multi-class classifying problem between multiple entities on the sentence level [24].

From studying learning curves, investigating the influence of the size of the training data on the performance of the models and statistically examine whether observed performance differences hold significance using the Mann-Whiteny U test, we find that BioRedditBERT and BERT are overall best for Reddit data (see Fig. 5 and Table 4). Furthermore, BioBERT and DistilBERT are most affected by the sample size whereas BioRedditBERT shows the smallest variation. In contrast, for PubMed, BioBERT is the best-performing model and ELECTRA is the most sensitive one to changing sample sizes (see Fig. 6 and Table 5). Still, all language models converge for larger sample sizes indicating that the size of the training data is sufficient to reliably learn the relation extraction task. It is interesting to note that the convergence for the Reddit data is smoother than for PubMed data, except for BioBERT, which means that the sample size of the training data could be chosen smaller than for PubMed to achieve a similar performance.

Aside from the convergence of the learning behavior, another important factor of a model is its runtime. We would like to emphasize that the execution time of the language models only includes the fine-tuning but not the pre-training. From Table 6 one can see that, overall, BERT and BioRedditBERT are by far the slowest models whereas DistilBERT and ELECTRA are the fastest. This is plausible since DistilBERT is a lightweight-version of BERT containing about 40% fewer parameters. Still, considering the absolute runtime of an hour, even BERT and BioRedditBERT can be fine-tuned without problems within a reasonable time.

Overall, from comparing the performance of the language models and their runtime one can conclude that each model is sufficient to learn COVID-related relations and the differences are only marginal.

Regarding the information content of the data provided by Reddit and PubMed, we found from Spearman's rank correlation tests that the correlation between entities for physical symptoms and the correlation between entities for mental symptoms is highly statistically significant. This indicates the rank order of the entities in PubMed and Reddit is very similar despite the different nature of these data sources. Taking into account the above results regarding the performance of the language models, our findings indicate the qualitative similarity of data from Reddit and PubMed. This opens up new possibilities for addressing future crises, especially in situations that demand prompt text analysis. The emphasis on utilizing data from social media platforms becomes crucial when high-quality information from scientific publications is not readily accessible.

Interestingly, from an additional analysis we found that the studied methods are not only capable of detecting known relations and entities but they are even capable of discovering new entities. Specifically, when looking into predicted entities, we find SARS-COV-19 (PubMed) as a synonym for COVID, palpitations, joint pain, chest pain, tightness, heart failure, loss of memory, hypertension, brain fog (Reddit and PubMed) for physical symptoms and Sinovac and Johnson and Johnson (Reddit) for vaccination synonyms. This capability is desirable because the specification of an exhaustive list of an entity can be very time-consuming or even impossible. Yet, as demonstrated by our analysis, the language models are still capable of identifying, at least some, of such inadequately represented relations in the training data.

The last question we address in this paper is the identification of misinformation. This question is inspired by the above findings about novel entities because the identification of relations that represent “misinformation” is inherently an ill-posed problem due to the fact that the establishment of a comprehensive list of misinformation is infeasible. Technically, we cast the identification of misinformation as a multi-class classification problem by extending our model to a 4 class problem where we consider the category “misinformation” as a new class. Since misinformation can appear in any scenario where information is transmitted, it is crucial to pay attention to this. For this reason, our current method is effective for a variety of texts but adapting it to other text types would require thoughtful consideration. To make sure the model functions widely, it should be thoroughly trained, evaluate its performance, and test it on a variety of datasets. We intend to implement this in the future to further improve and expand the capabilities of our approach.

From the analysis of misinformation, we observe that transformer models can effectively learn the task of misinformation detection, as evident from the individual F-score for the category “misinformation”, the macro-averaged F-score, and the AUC (see Table 10, Table 11). This demonstrates that transformer models not only have the capability to learn this task but also exhibit reliable proficiency in detecting misinformation. Considering that health-related misinformation has been identified as a severe problem on social media [59], [60], [61], our findings show that transformer models can provide an automatic approach to this problem.

Importantly, for this task, we utilized pre-trained models to reduce the training time, however, for the fine-tuning of the models, we used data that we manually annotated from Reddit and PubMed which is a very time-consuming task. Overall, this makes the detection of misinformation not a fully automated process. To overcome this limitation and achieve full automation in misinformation detection, we suggest that future studies explore semi-supervised and unsupervised learning techniques, along with leveraging pre-existing datasets. These approaches have the potential to further minimize manual efforts, improve scalability, and progress toward a fully automated model for detecting misinformation.

Taken together, all our results demonstrate that language models trained with data from PubMed or Reddit show very comparable results. This is certainly interesting because both data sources are quite different. Whereas the data from PubMed represent peer-reviewed scientific articles the data from Reddit correspond to informal discussions among people with diverse backgrounds and can be seen as “people's opinion”. Whether this similarity is a consequence of the editorial policies of Reddit or a self-organization process [62] is unclear. However, a consequence of this similarity is that for future epidemic events, there is no necessity to await the availability of peer-reviewed scientific publications, which can be a time-consuming process. Instead, data from social media can be employed to explore disease-related inquiries, offering a significant time advantage, a critical factor in the investigation of diseases such as COVID-19.

A final point we would like to highlight is that our analysis indicates that a moderate amount of data is sufficient for the reliable extraction of relations. Specifically, to achieve approximately 80% of its maximal performance in a three-class classification task, around 600 training samples are required, a finding consistent across both PubMed and Reddit datasets. Notably, for Reddit, this data can be swiftly gathered from social media within a few weeks. In summary, our results emphasize that social media data from Reddit serve as a valuable and high-quality resource for training relation detection models, and this is not substantially impeded by the presence of misinformation.

## 6 Conclusion

COVID-19 emerged as a global pandemic, posing unprecedented challenges to healthcare systems across the world. To establish effective countermeasures, it is crucial to gather data from diverse sources to enhance our understanding of the disease and derive evidence-based medical solutions. However, the process of generating such data is time-consuming, which adds further pressure on the healthcare system.

The findings in this paper support the usage of text data from the social media platform Reddit to extract COVID-related relationships. Using five transformer-based models, including BERT, BioBERT, and DistilBERT, we demonstrate that text data from PubMed, corresponding to peer-reviewed scientific publications, and Reddit, providing public discussions of layman, are qualitatively similar. This discovery has notable implications for future crises, as it underlines the advantage of utilizing text data from social media platforms. Unlike data obtained from PubMed, which undergoes a lengthy peer-review process, text data from social media can be gathered more rapidly. This expedited data acquisition enables quicker access to valuable information, especially during times of crisis.

Furthermore, we demonstrate that the transformer models can identify novel entities and relations not present in the training data and even reveal misinformation. While transformer-based models have been studied amply for diverse tasks, to our knowledge, we are the first to address these issues systematically. Also, we intend to implement semi-supervised and unsupervised learning techniques, along with leveraging pre-existing datasets in the future to further improve and expand the capabilities of our approach. Such approaches will reduce manual efforts, enhance scalability, and move towards a more fully automated model for detecting misinformation. In addition, misinformation can appear anywhere and to generalize our methodology, we intend to utilize this in various texts for instance from health, finance, politics, etc. In general, our approach opens up new possibilities for knowledge discovery and for addressing the spread of misinformation.

## CRediT authorship contribution statement

**Tanvi Sharma:** Writing – review & editing, Writing – original draft, Visualization, Methodology, Formal analysis, Data curation. **Amer Farea:** Writing – review & editing, Writing – original draft, Methodology. **Nadeesha Perera:** Writing – review & editing, Writing – original draft, Methodology. **Frank Emmert-Streib:** Writing – review & editing, Writing – original draft, Visualization, Supervision, Methodology.

## Declaration of Competing Interest

The authors declare that they have no known competing financial interests or personal relationships that could have appeared to influence the work reported in this paper.
